# Characterising the vertical separation of shale-gas source rocks and aquifers across England and Wales (UK)

**DOI:** 10.1007/s10040-018-1737-y

**Published:** 2018-03-20

**Authors:** Sian E. Loveless, John P. Bloomfield, Robert S. Ward, Alwyn J. Hart, Ian R. Davey, Melinda A. Lewis

**Affiliations:** 1 British Geological Survey, Maclean Building, Crowmarsh Gifford, Oxon OX10 8BB UK; 2 Environment Agency, Horizon House, Deanery Road, Bristol, BS1 5AH UK

**Keywords:** Shale gas, Vertical separation, Hydraulic fracturing, UK

## Abstract

Shale gas is considered by many to have the potential to provide the UK with greater energy security, economic growth and jobs. However, development of a shale gas industry is highly contentious due to environmental concerns including the risk of groundwater pollution. Evidence suggests that the vertical separation between exploited shale units and aquifers is an important factor in the risk to groundwater from shale gas exploitation. A methodology is presented to assess the vertical separation between different pairs of aquifers and shales that are present across England and Wales. The application of the method is then demonstrated for two of these pairs—the Cretaceous Chalk Group aquifer and the Upper Jurassic Kimmeridge Clay Formation, and the Triassic sandstone aquifer and the Carboniferous Bowland Shale Formation. Challenges in defining what might be considered criteria for ‘safe separation’ between a shale gas formation and an overlying aquifer are discussed, in particular with respect to uncertainties in geological properties, aquifer extents and determination of socially acceptable risk levels. Modelled vertical separations suggest that the risk of aquifer contamination from shale exploration will vary greatly between shale–aquifer pairs and between regions and this will need to be considered carefully as part of the risk assessment and management for any shale gas development.

**Electronic supplementary material:**

The online version of this article (10.1007/s10040-018-1737-y) contains supplementary material, which is available to authorized users.

## Résumé

Le gaz de schiste est considéré par beaucoup comme ayant le potentiel de procurer au Royaume Uni une plus grande sécurité énergétique, une croissance économique et des emplois. Cependant, le développement de l’industrie du gaz de schiste est fortement contesté en raison des problèmes environnementaux, qui comprennent le risque de pollution des eaux souterraines. Des données probantes suggèrent que l’isolation verticale entre les unités de schistes exploitées et les aquifères est un facteur important vis-à-vis du risque de contamination des eaux souterraines associé à l’exploitation du gaz de schiste. Une méthodologie est proposée pour évaluer la séparation verticale entre différents couples aquifères—schistes présents en Angleterre et Pays de Galle. L’application de la méthode est ensuite décrite pour deux de ces couples, d’une part l’aquifère du Groupe de la Craie du Crétacé et la formation des Argiles du Kimméridgien (Jurassique supérieur), et l’aquifère des Grés du Trias et la formation carbonifère des schistes du Bowland. Les enjeux de la définition de ce qui pourrait être considéré comme critère d’une « isolation sécuritaire » entre une formation à gaz de schiste et l’aquifère sus-jacent sont discutées, en particulier au regard des incertitudes sur les propriétés géologiques, l’extension des aquifères et la définition des niveaux de risques socialement acceptables. Les isolations verticales modélisées suggèrent que le risque de contamination d’un aquifère lié à une exploration du schiste variera grandement d’un couple schiste–aquifère à l’autre et d’une région à l’autre et que cela devra être examiné avec soin dans le cadre de l’évaluation du risque et de la gestion de tout développement du gaz de schiste.

## Resumen

Muchos consideran que el shale gas tiene el potencial de proporcionar al Reino Unido una mayor seguridad energética, crecimiento económico y empleos. Sin embargo, el desarrollo de una industria del shale gas es muy polémico debido a las preocupaciones ambientales, incluido el riesgo de contaminación del agua subterránea. La evidencia sugiere que la separación vertical entre las unidades de shale explotadas y los acuíferos es un factor importante en el riesgo para las aguas subterráneas en la explotación del shale. Se presenta una metodología para evaluar la separación vertical entre los diferentes pares de acuíferos y los shales que están presentes en Inglaterra y Gales. La aplicación del método se demuestra para dos de estos pares—el acuífero Chalk Group del Cretácico y la Kimmeridge Clay Formation del Jurásico Superior, y el acuífero de arenisca del Triásico y la Bowland Shale Formation del Carbonífero. Se discuten los desafíos para definir los que podrían considerarse criterios para una “separación segura” entre una formación de shale gas y un acuífero superpuesto, en particular con respecto a incertidumbres en las propiedades geológicas, extensiones de acuíferos y la determinación de niveles de riesgo socialmente aceptables. Las separaciones verticales modeladas sugieren que el riesgo de contaminación del acuífero por la exploración del shale variará mucho entre los pares de shale–acuífero y entre regiones, y esto deberá considerarse cuidadosamente como parte de la evaluación y gestión de riesgos para cualquier desarrollo de shale gas.

## 摘要

许多人认为页岩气具有给英国提供较大能源安全、经济增长和工作的潜力。然而,由于环境方面的关切包括地下水污染的风险,页岩气行业的发展争议很大。证据表明,开采过的页岩单元和含水层之间的垂直分离是页岩气开采对地下水产生的风险中一个重要的因素。这里论述了评价了英格兰和威尔士现有的不同对含水层和页岩之间垂直分离的方法。然后通过展示了其中的两对—白垩纪白垩组含水层和上侏罗纪Kimmeridge粘土层、三叠纪砂岩含水层和石炭纪Bowland页岩层介绍了该方法的应用情况。论述了在定义什么可能是页岩气层和上覆地层之间“安全分离”的标准中的挑战,特别是有关地质特性的不确定性、含水层范围和社会接受风险水平的确定等。模拟的垂直分离表明,由于页岩勘探导致的含水层污染风险每对之间、不同地区之间差异很大,在任何页岩气开发中这需要作为风险评价和管理的一部分认真给予考虑。

## Resumo

O gás de xisto é considerado um potencial fornecedor de maior segurança energética, crescimento econômico e empregos ao Reino Unido. Porém, o desenvolvimento de uma indústria de gás de xisto é altamente controverso devido a preocupações ambientais, como o risco de poluição das águas subterrâneas. Evidências sugerem que a separação vertical entre unidades de xisto exploradas e aquíferos é um fator importante de risco para as águas subterrâneas na exploração de gás de xisto. Uma metodologia é apresentada para avaliar a separação vertical entre diferentes pares de aquíferos e blocos de xisto presentes em toda a Inglaterra e País de Gales. A aplicação do método é feita em dois pares—o Aquífero do Grupo Cretáceo Chalk com a Formação Argilosa do Jurássico Superior Kimmeridge, e o Aquífero de Arenito Triássico com a Formação Xistosa do Carbonífero Bowland. Os desafios para definir o que pode ser considerado critérios de “separação segura” entre uma formação de gás de xisto e um aquífero sobrejacente são discutidos, em particular no que diz respeito a incertezas em propriedades geológicas, extensões de aquíferos e determinação de níveis de risco socialmente aceitáveis. As separações verticais modeladas sugerem que o risco de contaminação do aquífero por exploração de xisto variará muito entre os pares de xisto–aquífero e suas regiões, devendo assim serem consideradas cuidadosamente como parte da avaliação e gerenciamento de risco para qualquer desenvolvimento de gás de xisto.

## Introduction

**Fig. 1:**
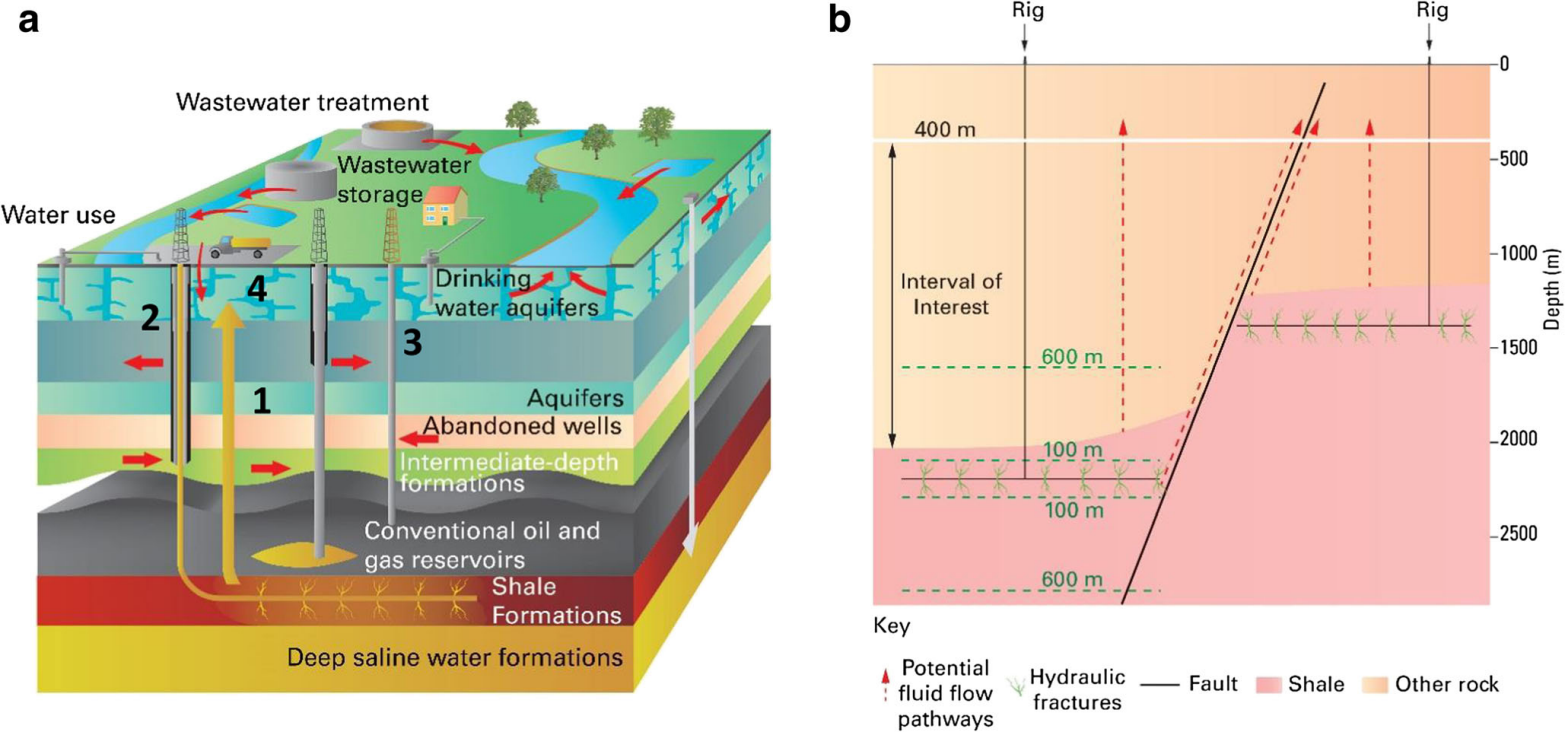
**a** Schematic diagram showing potential source-pathway-receptors resulting from shale gas exploration and production (after Vengosh et al. 2014). Numbers correspond to pathways described in the text. Diagram not to scale. **b** Schematic diagram illustrating the effect of aquifer–shale separation distances and potential direct pathways through the intervening interval, including migration through the intervening rock mass, and aquifer and hydraulic fractures linking with a permeable fault zone. Green dotted lines illustrate 100 and 600-m hydraulic fracture heights

Increasing demands for use of the deep subsurface, such as for storage of nuclear waste materials, sequestration of CO_2_, and the development of conventional and unconventional on-shore hydrocarbon resources can place additional pressures on groundwater resources. One such use of the subsurface, the production of natural gas from shales, or shale gas, has received growing attention in the last few years, with recognition of the range of potential threats to groundwater (e.g. Jackson et al. 2014). The industry has expanded dramatically since the year 2000 in North America due to cost effective extraction technologies including directional drilling and slick water fracking fluids (US EPA 2016; Gallegos and Varela 2015). Shale gas exploration and production is now taking place in a number of other countries around the world, including Europe (e.g. Scotchman 2016). Low-level resource-assessment activity has been ongoing in the UK since 2011, with two hydraulic fracturing licences now granted. Groundwater is an important resource in England and Wales, providing an average of 31% of water resources, and up to 100% in some areas of southeast England. Pressures on groundwater resources from the development of shale gas may include water supply issues associated with drilling and hydraulic fracturing of wells (Flavin and Kitasei 2010; Gregory et al. 2011; Wood et al. 2011; Stuart 2012; Vengosh et al. 2014; Kondash and Vengosh 2015). Potential groundwater contamination has been postulated from a range of possible contamination pathways illustrated in Fig. 1a, including: (1) migration of produced gases and/or fluids used in the hydraulic fracturing process though rock units separating shale-gas source rocks and overlying aquifers (Vengosh et al. 2014; Myers 2012a); (2) contamination via defective production wells; (3) leakage of abandoned wells (Davies et al. 2014); and (4) spills of chemicals, flow back and produced waters at the land-surface that could percolate to shallow aquifer systems (Vengosh et al. 2014; Rozell and Reaven 2011).

Potential scenarios for migration of contaminants from shales to aquifers (pathway 1) are shown in Fig. 1b. The greater the vertical separation between the shale and overlying aquifer, the more likely the intervening rock mass will limit the upward migration of fluids and gases and thereby reduce the risk of contamination of groundwater associated with this pathway. There will also be a further reduction of risk when rock units with low permeability are present within the overlying rock mass, contributing to a relatively low bulk vertical hydraulic conductivity (Freeze and Cherry 1977), and also where there is an absence of through-going, conductive fracture networks which might connect them (Myers 2012a; Cai and Ofterdinger 2014).

A methodology is presented here for evaluating the vertical separation between shales and aquifers across England and Wales. Its application is demonstrated for two important principal aquifer–shale combinations—the Cretaceous Chalk Group aquifer and the Kimmeridge Clay Formation, and the Triassic sandstone aquifer and the Bowland Shale Formation (the upper shale within the Craven Group). The Chalk Group and the Triassic sandstone aquifers are the most important aquifers for water supply in southern and northern England respectively (Allen et al. 1997), while the Bowland Shale Formation is currently the principal target for shale gas exploration in England, with planning permission having been recently granted for hydraulic fracturing operations (DCLG 2016).

### Context: the concept of ‘safe separation’

A number of studies have sought to quantify a ‘safe separation’ distance between the zone of hydraulic fracturing and overlying aquifers (Davies et al. 2012; Kissinger et al. 2013); however, despite an increasing body of research there is a lack of consensus regarding what might be considered a ‘safe separation’ on the basis of being confident that the risks will be acceptable, i.e. be extremely low. The term itself currently has no commonly agreed meaning and is a relative rather than absolute concept that encompasses considerations of the value of groundwater resources within a particular society and the consequent nature of risk assessment undertaken within widely varying regulatory frameworks (Alberta Energy Regulator 2013; Environment Agency 2013a). A useful way, therefore, of using the concept of ‘safe separation’ is to aim for a separation distance over which no contaminant breakthrough would be expected to occur but in the unlikely event that it did, the concentrations would be so low that they would not be harmful or of concern to humans and the environment (Myers 2012a). Therefore information about contaminant concentrations at source, timescales of interest in relation to contaminant attenuation, the physical proximity of sources and receptors, and the physical properties of the intervening interval are all relevant to the concept of a ‘safe separation’ (Birdsell et al. 2015). The present study addresses methods to constrain uncertainty about the separation of sources and receptors and looks at the impacts on the available area of potential shales if suggested safe separation distances are applied. It does not attempt to define safe separation due to the large number of other factors that would need to be considered.

Vertical separation distances between aquifers and shales vary greatly depending on the depositional and tectonic setting and resultant relative position of aquifers and shales in the local stratigraphy. Unlike existing plays in North America, where shale gas plays and overlying aquifer units are typically regionally extensive and often in relatively simple structural settings, in many parts of Europe, including the UK, the geological and hydrogeological settings may be much more complex (Ward et al. 2015). For example, in the UK there can be multiple potential shale gas targets within relatively complex stratigraphic sequences and structural settings that may alter the spatial relationships between shales and aquifers across a region (Andrews 2013). In these complex geological settings, knowledge of aquifer–shale separation and its variability will be a critical consideration for well-regulated, future development of shale gas resources. Consequently, there is a need for high-level regional-scale screening tools such as the one described here, to characterise and investigate the vertical separation between major shales and aquifers.

## Data and methods

Consistent with the source-pathway-receptor conceptual framework used by the Environment Agency (England; Defra 2011), hydraulic fracturing of shales is considered as a potential source of contamination (Jackson et al. 2013), aquifers as potential receptors, and the intervening volume of rock and associated discontinuities the potential pathway. The aim of the current study is to develop an approach to estimate and map spatial variations in the distance (vertical separation) between identified receptors and sources at a regional to national scale. To do this there is a need to identify the aquifers (receptors) and shales (sources) of interest across the area of interest (England and Wales) and then to define their respective boundaries, in this case, the base of the aquifer and top of the shale.

**Fig. 2:**
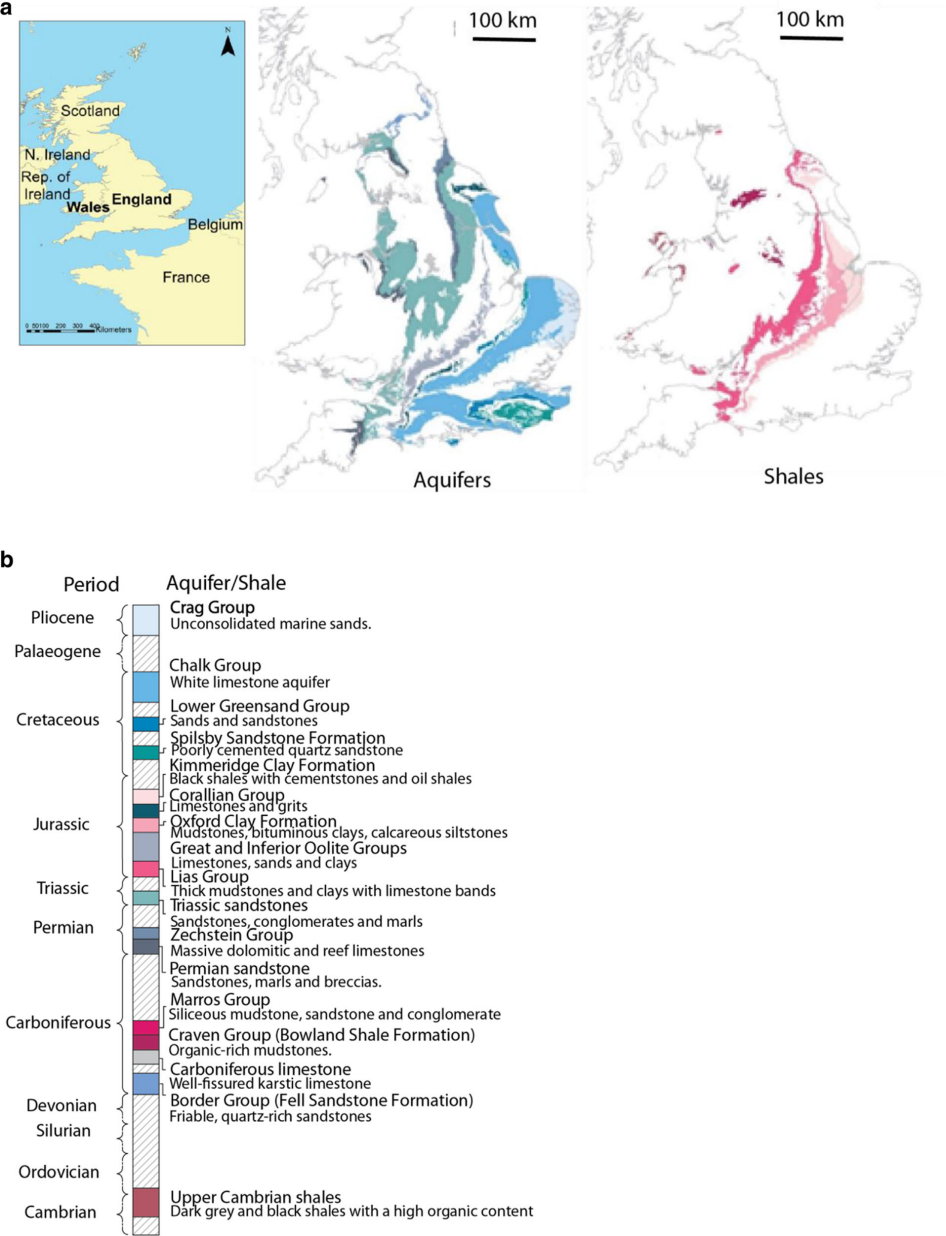
**a** Principal aquifers and major shales at outcrop in England and Wales (part of the United Kingdom). Rock units are generally younger towards the southeast. Note the principal aquifers are extensive at outcrop, while the shale units are more extensive in the subsurface than at outcrop. **b** Schematic stratigraphy of these units (scaled to time not unit thickness). For more detail about the aquifer and shale units see Tables S1 and S2 of the electronic supplementary material (ESM)

In England and Wales, principal aquifers are defined as ‘geological strata that exhibit high permeability and usually provide a high level of water storage’. Also ‘they are capable of supporting water supply at a strategic scale and are often of major importance to river baseflow’ (EA 2013a). Principal aquifers provide most of the potable groundwater supply across England and Wales and are therefore the focus of the present study. The Environment Agency for England and Natural Resources Wales recognise 11 main bedrock principal aquifers. The outcrop pattern of these is shown in Fig. 2a and their relative stratigraphic position shown in a schematic column in Fig. 2b.

Principal aquifers are defined in terms of geological strata (EA 2013b). As the base of these aquifers is considered to be co-incident with the base of the geological unit this means that the base of aquifers can be up to at least 7 km bOD (below Ordnance Datum, or sea level)—much deeper than the maximum depth of exploitation, given that generally water quality and groundwater yields reduce significantly with depth (Allen et al. 1997).

In the UK, environmental objectives for groundwater (quality and quantity) are established as part of the EU Water Framework Directive and applied to groundwater bodies. The UK Technical Advisory Group (UKTAG) have issued guidance on the definition and delineation of groundwater bodies (UKTAG 2011), recommending a default maximum thickness for bedrock groundwater bodies of 400 m, unless local knowledge indicates that a different depth should be applied.

The UKTAG recommendation is consistent with the zone of active exploitation of groundwater in England based on the distribution of borehole depths within a given aquifer. Most exploitation of groundwater takes place at depths of a few tens of metres below groundwater level—Fig. S1 of the electronic supplementary material (ESM). However, different aquifers are exploited to different maximum depths (dependent on their respective depth-yield relationships)—for example, Triassic sandstones are exploited to much greater depths (a number of boreholes exceed 400-m depth) than the Chalk Group (maximum depth ~200 m). Consequently, for the purposes of the separation analysis, and consistent with the UKTAG recommendations, it was assumed that the base of a given principal aquifer is either the base of the geological unit forming the aquifer, or if the unit is present at greater than 400 m below ground level (bgl), the base of the aquifer is assumed to be 400 m bgl.

The first extensive review of shale gas prospectivity in the UK (Smith et al. 2010) identified potential shale gas targets as the main organic-rich black shales from Cambrian to late Jurassic age, which could have reached the thermogenic gas window. Using this and related work, reports for the Dept. of Energy and Climate Change (Andrews 2013; DECC 2012; Andrews 2014) highlighted the following six units as potential shale-gas source rocks: the Kimmeridge Clay Formation; Oxford Clay Formation; Lias Group; Marros Group; Bowland Shale Formation; and the Upper Cambrian shales. These units are used in this study and the outcrop of the shales is shown in Fig. 2a, while Fig. 2b shows their position in a schematic stratigraphic column.

In this study, the top of shale units has been identified for the purposes of calculating separations between shales and aquifers. In reality, the potential target or ‘sweet zone’ for hydraulic fracturing and shale gas production will be below this level, within the body of the shale (DECC 2012; Andrews 2014); however, this information is not yet consistently available at the regional- to national-scale, and hence a precautionary approach has been adopted. Since the maximum depth of principal aquifers considered here is 400 m bgl and the shale units are unlikely to present a commercial prospect shallower than this level (and in reality legislation prevents high volume hydraulic fracturing above 1,000 m bgl) the only scenarios considered are those where principal aquifers overlie shale units.

## Analysis of aquifer–shale separation based on a three-dimensional geological model

The British Geological Survey (BGS) National Geological Model (NGM) of Great Britain (Mathers et al. 2012, 2014a, b) was used as the basis for modelling the aquifer base and the top of the shale in England and Wales. The NGM is a digital model, developed using the geological modelling software GSI3D (Mathers et al. 2014b). It consists of a series of geological sections (‘fences’) across the UK, typically with spacing of about 30 km and to a depth of up to 5 km. It is built on a common stratigraphic succession for the UK. Depending on the underlying geological data for each section, the location of geological boundaries in each section may have a vertical accuracy of between about 10 and 100 m.

**Fig. 3:**
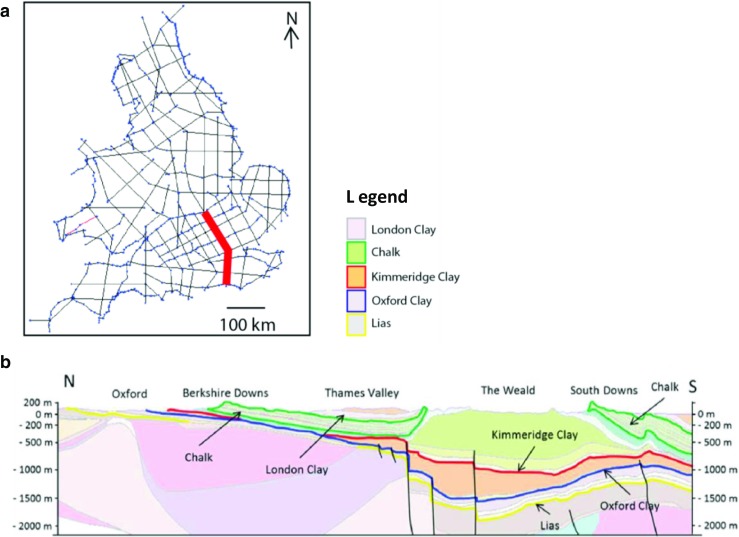
**a** Map illustrating the location of the geological sections from the BGS National Geological Model (NGM) used in this work. The section shown in (**b**) is highlighted in red. **b** Representative cross section from the NGM, through central southern England and the western end of the Weald. The Chalk Group aquifer is outlined in green, and upper surfaces of the Kimmeridge Clay Formation, Oxford Clay Formation, and Lias Group are highlighted by red, blue and yellow lines respectively

A subset of 84 geological cross sections across England and Wales, totalling ~12,000-km line length, was used to construct the aquifer and shale surfaces of interest (Fig. 3a; Mathers et al. 2014a). Top or base surfaces were generated by applying a simple linear interpolation algorithm between polylines along geological sections and the intersection of the land surface and outcrop. A 3 km × 3 km-grid resolution was used for the interpolation of the surfaces reflecting the degree of uncertainty in the position of surfaces between cross-sections, while still honouring the overall depth distribution data. Where principal aquifers were present below 400 m, the base of these units was modified to show a maximum depth of 400 m bgl. Where principal aquifers were underlain by shale units, spatial queries in ArcGIS were used to calculate vertical separations between the pairs of top shale and base aquifer surfaces.

## Results

**Fig. 4:**
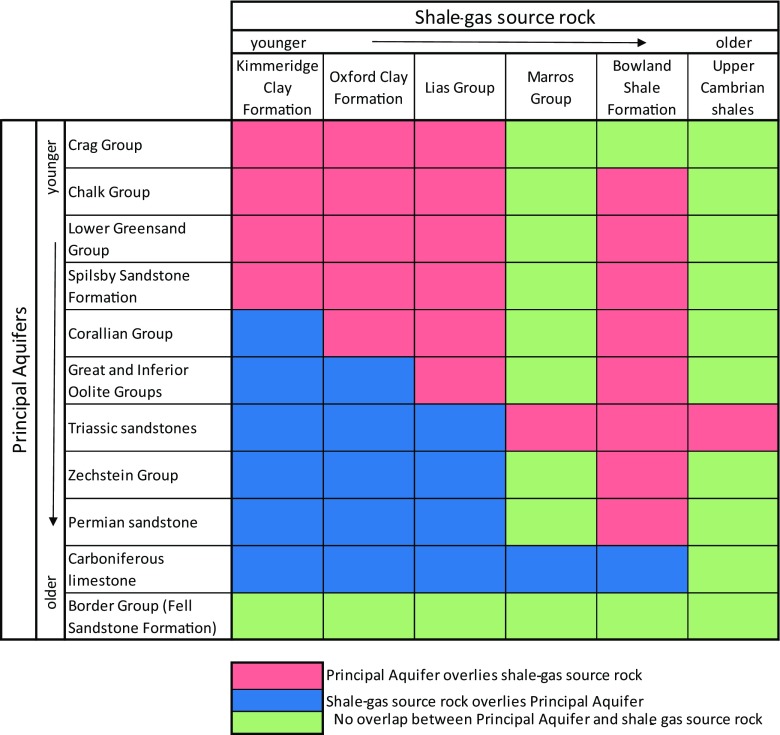
Matrix illustrating the large number of spatial relationships between the principal aquifer and shale units in England and Wales due to the complex geology and structural history

**Fig. 5:**
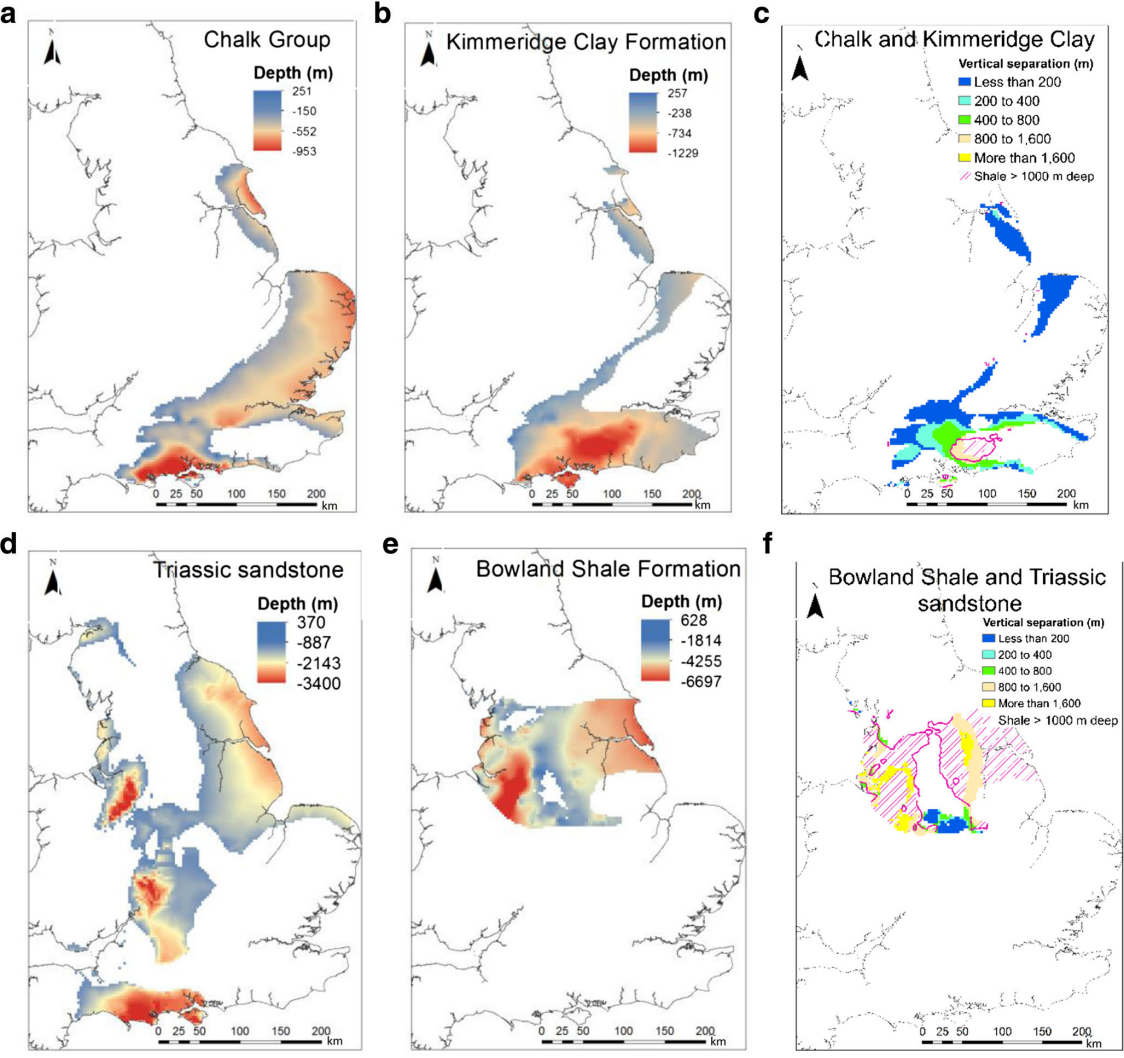
Maps showing examples of modelled outputs. Depth is meters above or below Ordnance Datum. **a** Base of the Chalk Group aquifer, **b** top of the Kimmeridge Clay Formation, **c** Chalk Group and Kimmeridge Clay Formation vertical separation, **d** base of the Triassic sandstone aquifer, **e** top of the Craven Group (Bowland Shale Formation), **f** Triassic sandstone and Craven Group (Bowland Shale Formation) vertical separation. Contours and shading (**c** and **f**) show the areas where the shale formation is >1,000 m bgl

Figure 4 shows that there are 25 pairs of surfaces where major shales underlie principal aquifers in England and Wales. The complexity of some of these relationships in the geological sequence can be seen along a representative section from central England through the western end of the Weald (Fig. 3b), highlighting the spatial relationships between the Chalk Group aquifer and three different shales, the Kimmeridge Clay Formation, the Oxford Clay Formation and the Lias Group. Figure 5 shows the modelled full crops and vertical separation for the Chalk Group principal aquifer and the Kimmeridge Clay Formation and the Triassic sandstone principal aquifer and the Craven Group (Bowland Shale Formation).

**Fig. 6:**
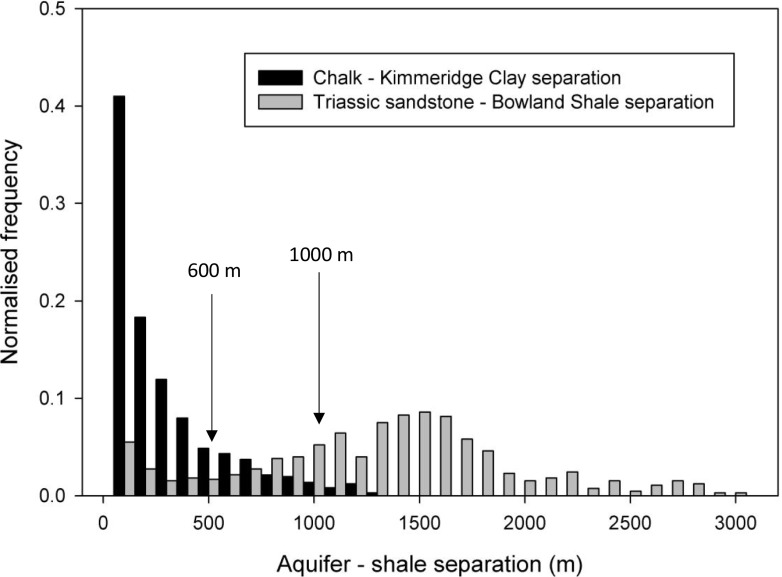
Distribution of vertical separation for the two aquifer–shale separation map examples shown in Fig. 5; 600 m and 1,000 m represent the maximum height of induced hydraulic fractures and natural hydraulic fractures (e.g. Davies et al. 2012)

Vertical separations are typically smaller between the Chalk Group and Kimmeridge Clay Formation than the Triassic sandstone and Bowland Shale Formation pair (Fig. 5; Tables 1 and 2), reflecting the proximity of the Chalk Group and Kimmeridge Clay Formation in the stratigraphic sequence over much of England. In addition, in southern and eastern England regional structures are typically relatively simple with more gentle, open fold structures compared with deeper, fault controlled basins and tighter, localised folding in central and northern England. Consequently, the Triassic sandstone and Bowland Shale Formation separation map (Fig. 5f) shows more spatially complex distributions of vertical separation than between the Chalk Group and Kimmeridge Clay Formation pair (Fig. 5c). These differences are also reflected in the respective histograms of relative frequency of vertical separations,(Fig. 6); however, a common feature of both separation maps is that these aquifers only cover part of the respective shales, 51% in the case of the Chalk Group and Kimmeridge Clay Formation and 26% in the case of the Triassic sandstone and Bowland Shale Formation pairs. This area is reduced further when considering the minimum permitted depth of high volume hydraulic fracturing in England and Wales, shown by the 1,000-m contour of the shales (Figs. 5c,f), to 2 and 16% respectively. Full crop maps of all the principal aquifers, shales and the respective separation maps are available in the supporting information (Fig. S2 of the ESM), and from the BGS website (BGS 2016).

**Table 1:** Summary statistics of vertical separations for individual aquifers and shales. *OD* relative to Ordnance Datum; *SD* standard deviation

Aquifer or shale	Area (km^2^)	Min. depth (m OD)	Max. depth (m OD)	Mean depth (m OD)	SD of depth data (m)
Chalk Group	33,165	251	−953	−181	183
Kimmeridge Clay Formation	23,670	257	−1,229	−351	320
Triassic sandstone	44,930	370	−3,400	−667	605
Bowland Shale Formation	24,120	628	−6,697	−1,539	1,097

**Table 2:** Summary statistics of vertical separations for both pairs of aquifers and shales. *OD* relative to Ordnance Datum; *SD* standard deviation

Aquifer/shale pair	Area (km^2^)	Min. separation (m)	Max. separation (m)	Mean separation (m)	SD of separation data (m)
Chalk Group/Kimmeridge	12,996	0	1,239	229	259
Triassic sandstone/Bowland Shale Formation	6,255	0	3,935	1,207	746

## Discussion

### Validation of mapped surfaces

Validation of the base aquifer or top shale surface maps can be undertaken if independent mapping of these surfaces is available; however, there are very limited data available for most of the surfaces. One that has been mapped is the Bowland Shale Formation across northern England (Andrews 2013). The top surface of the Bowland Shale Formation produced in this study was compared with a map of the depth to the top of the Bowland-Hodder shale unit produced by interpretation of commercially available seismic sections for the region, which incorporated information on fault distributions and throws. A comparison of the two showed that, despite the limited number of sections used to create the surface, the relatively coarse grid size and simple interpolation method used, and the lack of information about fault control, the match between the two outputs from the different approaches is considered to be very good (see Fig. S3 of the ESM).

### Implications for the concept of ‘safe separation’ in England and Wales

Despite an increasing body of research there remains a great deal of uncertainty surrounding definition and quantification of safe separation distances (Rozell and Reaven 2011; Vengosh et al. 2014). The following discussion summarises the evidence for safe separation distances associated with different migration pathways (rock matrix, induced and natural fractures) from geochemical, mechanical and numerical modelling studies and discusses possible physical limits or thresholds that can guide decision making.

Upward migration of deep brines (e.g. Warner et al. 2012; Llewellyn 2014) and gases (e.g. Molofsky et al. 2013; Moritz et al. 2015) with vertical migration over distances of up to 2,400 m (Llewellyn 2014) has been reported in shale basins, unrelated to human activity. In such cases faults and fractures are often thought to act as preferential pathways to subsurface flow (Warner et al. 2012; Molofsky et al. 2013; Llewellyn 2014; Moritz et al. 2015); however, a driving force is also required for brine migration, such as a deep hydrodynamic pressure creating an upwards hydraulic gradient (Warner et al. 2012). Neither the timescales of these processes, nor the possible influences from shale gas exploitation are well constrained. Geochemical data can provide evidence for such contamination as well as information regarding sources, pathways and timescales; however, tracing potential contamination from shale gas operations at depth is challenging due to the relatively short time frame since the start of exploitation compared to typically very low groundwater flow rates both at depths at which hydraulic fracturing takes place and in the overburden (Vengosh et al. 2014), in addition to a general lack of pre-operation geochemical baseline data.

Whilst there may be a significant number of potential contaminants released or mobilised at depth, methane can be considered a key indicator of contamination because of its buoyancy (Vengosh et al. 2014). Its migration can also continue for longer after the fracking process since large upwards driving forces are not required (Kissinger et al. 2013). Methane can be thermogenic, formed through high temperatures and sourced from hydrocarbon reservoirs including shales, and is a common natural component of groundwater in many areas overlying shale reservoirs (Moritz et al. 2015; Li et al. 2016); however, it may also be biogenic, being produced by bacterial decomposition of organic material at shallower depths. Baseline studies across Great Britain have found that methane is widely present in groundwater (Gooddy and Darling 2005; Darling and Gooddy 2006; Bell et al. 2016); therefore, its use as an indicator needs to be carefully considered. The source of methane can be distinguished using methods such as stable isotope analyses (e.g. Osborn et al. 2011; Moritz et al. 2015). Other tools such as noble gas, volatile organic compounds and component ratio analysis can also help to determine the source (Di Giulio et al. 2011; Osborn et al. 2011; Darrah et al. 2014; Llewellyn et al. 2015; Wen et al. 2016). In most cases, especially where gases have migrated over large distances, a combination of tools should be used as reliance on a single tool can be misleading.

A number of studies have reported groundwater with higher methane concentrations close to shale-gas exploitation activities. Higher isotopic ratios, low methane to higher-chain hydrocarbons ratios and associated noble gases, indicate a thermogenic origin for the methane (Di Giulio et al. 2011; Osborn et al. 2011; Darrah et al. 2014; Llewellyn et al. 2015). Faulty well casing rather than upward migration through the bedrock is generally considered as the most likely source of contamination (Osborn et al. 2011; Darrah et al. 2014).

The only known case of contamination by hydraulic fracturing chemicals is at Pavillion, Wyoming (Di Giulio et al. 2011; Wright et al. 2012; Di Giulio and Jackson 2016; US EPA 2016), although it is also suspected above the Marcellus shale in Pennsylvania (Llewellyn et al. 2015). The Wind River Formation is the principal source of groundwater in the Pavillion area but the same formation is also one of the main gas targets (Di Giulio et al. 2011). Contamination is thought to have occurred because stimulation fluids were directly injected into water-bearing units, but there was also casing failure at five production wells, which could allow migration into water-bearing units (Di Giulio and Jackson 2016). These scenarios were compounded by the shallow (often <500 m and a minimum of 323 m) depth of hydraulic fracturing, with very limited vertical separation compared to the depth of domestic water wells (up to 229 m deep; Jackson et al. 2013; Di Giulio and Jackson 2016) and lack of any intervening hydraulic barrier (Di Giulio et al. 2011). Above the Marcellus shale, Llewellyn et al. (2015) suggest that contaminants were transported along natural fractures which intersected wells, during injection; however, it should be noted that other studies have not found a relationship between increased methane and proximity to oil and gas wells (e.g. Molofsky et al. 2013; Li and Carlson 2014; Wen et al. 2016).

The presence of preferential pathways such as permeable faults, significantly increases the likelihood (decreases travel time and/or increases distance over which migration might occur) of contaminants migrating between shales and aquifers (Myers 2012a; Gassiat et al. 2013; Kissinger et al. 2013; Lange et al. 2013; Cai and Ofterdinger 2014; Reagan et al. 2015). Numerical modelling by Reagan et al. (2015) found that while gas breakthrough time was roughly an order of magnitude greater when the vertical separation was quadrupled, permeability and overall volume of the connecting fault or fracture, and production characteristics were found to have a greater impact than separation distance.

Importantly, these numerical models of contamination transport do not consider the likelihood of particular geological conditions existing (Reagan et al. 2015) and should be regarded in the context of their assumptions—both in terms of hydrogeological conditions and shale gas operations (Myers 2012a, b; Saiers and Barth 2012). Site-specific models will provide a much better indication of risks than generalised models. Only one contaminant transport model thus far has been produced for UK shales, representing the Bowland Shale Formation and overlying sequence, including the Sherwood Sandstone Group (part of the Triassic sandstone) aquifer (Cai and Ofterdinger 2014). Based on this model, the authors suggest that hydraulically fractured Bowland Shale Formation with a vertical separation of 1,600 m is unlikely to pose a risk to the overlying groundwater when the induced hydraulic fracture aperture is <0.2 mm. However, where modelled induced fracture apertures were greater, upwards fluid transport was found to be very sensitive to fracture height, and the upward chloride mass flux could potentially pose a risk to the overlying aquifers in as little as 100 years where induced factures intercepted a fault connecting the Bowland Shale Formation and Sherwood Sandstone aquifer. Vertical transport of fluids was also found to be sensitive to hydraulic properties of the intervening aquifers (Cai and Ofterdinger 2014).

The Bowland Shale Formation at Preese Hall, Lancashire was the first shale gas site in the UK to be hydraulically fractured in 2011, but the test was stopped when it was found to induce seismicity. Seismic sections from this site show that the Bowland Shale Formation is heavily fractured and faulted, although the faults in the immediate vicinity are relatively small and contained within rocks of Carboniferous age or the “impervious” unit above it. However, there are larger faults in the wider area such as the Thistleton Fault, which reaches the St Bees Sandstone (basal part of the Triassic sandstone; De Pater and Baisch 2011).

The aforementioned studies show that, where present, the extent of fractures and faults are considered important for contaminant migration between exploited shales and aquifers. The height of potential fractures and their potential to link shales and aquifers should be a primary consideration when considering safe vertical separation distances. While geophysical data can be used to image fracture height in the subsurface (Davies et al. 2012; Fisher and Warpinski 2012) data availability remains relatively limited—for example, only 0.5% of the hydraulic fracturing operations in the 100 hydraulic fracturing studies in “The Well File Review” were monitored with seismic arrays (US EPA 2016). Nevertheless, studies assessing induced fracture height from microseismic and micro-deformation data indicate that most hydraulic fractures are less than 100 m in height (Davies et al. 2012; Fisher and Warpinski 2012) and <1% of hydraulic fracturing stages have fractures greater than 350 m in height (Davies et al. 2012). Upper bounds to the height of induced hydraulic fractures are estimated to be between 460 and 588 m in height (Davies et al. 2012; Fisher and Warpinski 2012; Kim and Moridis 2015). Figure 1b shows 100 and 600 m hydraulic fracture heights to provide an indication of this scale. Natural hydraulic fracture pipes are found to be slightly longer, with most between 200 and 400 m in height (33% > 350 m in height), and up to a maximum of ~1,106 m, possibly resulting from the greater fluid volumes involved and occurrence in more extensively homogeneous lithologies (Davies et al. 2012; Lacazette and Geiser 2013). The height of induced fractures is likely to vary from site to site as the propagation of fractures can be impeded by the nature of certain overlying geological units which act as barriers (De Pater and Baisch 2011; Fisher and Warpinski 2012; Kim and Moridis 2015) and operational parameters such as injection fluid pressure and volume (Flewelling and Manu 2013; Kim and Moridis 2015). The longest induced fractures are thought to result from interactions with existing faults (Davies et al. 2012). Monitoring of shale exploitation in Greene County, Pennsylvania found the maximum height of hydraulically induced fractures measured by microseismicity corresponds with the maximum height of faults in the region (Hammack et al. 2014). It should be pointed out that it is not clear that fracture apertures of hydraulic significance can be sustained at depth once the hydraulic fracturing fluid overpressure has been removed and formation pressure is reduced below hydrostatic once a well is producing (Stokstad 2014). In addition, Fisher and Warpinski (2012) argue that while induced fractures are predominantly vertical at depths greater than ~1,200 m, at depths shallower than ~ 600-m-induced fractures would predominantly be horizontal due to a decrease in overburden stress. Therefore, since the default maximum depth of groundwater bodies in the UK is 400 m (UKTAG 2011), vertical fractures might not directly extend to aquifers from depth.

### Implications

The preceding evidence suggests that the risk of contamination to aquifers from shale gas operations increases with reduced vertical separation distances between the exploited shale and aquifer. The presence of preferential flow paths from induced or natural fractures will increase the vertical separation that can be considered safe, but the risk decreases strongly with increased vertical separation due to both fracture (Davies et al. 2012; Fisher and Warpinski 2012) and fluid migration (Reagan et al. 2015) characteristics. Given this, and the large variations in vertical separation between the shale and aquifer units presented in this work—from 0 m for the Chalk Group and Kimmeridge Clay Formation to over 3,000 m for the Triassic sandstone and Bowland Shale Formation (Figs. 5 and 6; Tables 1 and 2), it is inferred that there are likely to be large disparities in the possible risk to aquifers from shale exploitation in the UK.

The impacts on the area and location of shales that might remain exploitable by applying thresholds for vertical separation with the respective aquifers (Davies et al. 2012; Fisher and Warpinski 2012; Kim and Moridis 2015; Lacazette and Geiser 2013) are shown in Fig. 6. In all, 80% of the overlap of the Bowland Shale Formation and Triassic sandstone aquifer and 11% of the Kimmeridge Clay Formation and Chalk Group aquifer have vertical separations greater than 600 m, which is reduced to 64% of the Bowland Shale Formation and 3% of the Kimmeridge Clay Formation if a threshold of 1,000-m vertical separation is applied.

Amendments to the UK Petroleum Act 1998 in the Infrastructure Act (2015) establishes a minimum distance below ground level of 1,000 m at which high volume hydraulic fracturing may be carried out (Fig. 5c,f). This distance is extended to 1,200 m for protected groundwater source areas (source protection zone 1) and other protected areas—“The Onshore Hydraulic Fracturing (Protected Areas) Regulations” (2016). There would therefore be a minimum distance of 600 m from the default maximum depth of the base of the groundwater body defined by UKTAG (2011) and a shale unit undergoing high volume hydraulic fracturing and a minimum distance of 800 m for protected areas.

## Conclusions

This work provides an initial overview of the co-location of the main shales and principal aquifers in the UK. The mapped outputs are from aquifer–shale pairs, but it should also be noted that multiple aquifers and/or shales are common. The use of all existing seismic and borehole data, in addition to refinement of the aquifer and shale surfaces would improve accuracy of vertical separation estimates. Since pre-existing preferential pathways may present greater risk, it would be useful to assess fault height and pervasiveness in conjunction with shale-aquifer vertical separation, in addition to the hydraulic properties of the intervening units. This would only be a first step to assessing the risk to aquifers from potential shale exploitation and would need to be followed by in-depth site-specific assessments taking additional data and local factors into account.

The vertical separation distances of two of the aquifer–shale pairs (Chalk Group-Kimmeridge Clay Formation and Triassic sandstone-Bowland Shale Formation) out of a possible 25 aquifer–shale combinations identified in the UK have been presented and discussed. In general, the vertical separation for the Chalk Group and Kimmeridge Clay Formation is much smaller than for the Triassic sandstone and Bowland Shale Formation though both pairs show quite large variability, reflecting the complex geological history and basin development of the UK. Safe vertical separation distances are difficult to determine due to large uncertainties in geologic parameters and determination of socially acceptable risk levels and aquifer depth limits. Modelled vertical separations suggest that the risk of aquifer contamination from shale exploration will vary greatly between shale–aquifer pairs and between regions.

## Electronic supplementary material

ESM 1 (PDF 1622 kb)
