# Supplementary material for: Characterising the vertical separation of shale-gas source rocks and aquifers across England and Wales (UK)
Source: Hydrogeol J. 2018 Mar 20;26(6):1975–87. doi: 10.1007/s10040-018-1737-y (PMC6417439; doi:10.1007/s10040-018-1737-y)
Supplement: Supplementary file 1 — (PDF 1622 kb) [file 10040_2018_1737_MOESM1_ESM.pdf]

Hydrogeology Journal — Electronic Supplementary Material

Characterising the vertical separation of shale-gas source rocks and aquifers across England and Wales (UK)

Sian E. Loveless[1*], John P. Bloomfield[1], Robert S. Ward[1], Alwyn Hart[2], Ian Davey[2], Melinda Lewis[1]

[1]British Geological Survey, Maclean Building, Crowmarsh Gifford, Oxon, OX10 8BB, UK

[2]Environment Agency, Horizon House, Deanery Road, Bristol. BS1 5AH.

*Corresponding author: Sian Loveless, sian@bgs.ac.uk

Table S1. List and description of the principal aquifers in England and Wales analysed in this work.

Principal aquifer	Description of the principal aquifer
Crag Group	The Crag Group is a locally important aquifer, up to about 70 m thick, found in parts of East Anglia. It generally consists of unconsolidated marine sands that may be locally hard and consolidated. The yield is typically moderate to low, averaging about 3 l/sec and up to 11 l/sec. Water is iron-rich and hard.
Chalk Group	The Chalk Group is the major aquifer of southern and eastern England. It is a dual porosity, dual permeability white limestone aquifer. Yields are highest (up to 150 l/sec) where the density of fractures is greatest and/or where the fractures are enlarged (sub-karst) typically in the top 80 to 100 m of the aquifer. Porewater at depth may be saline where the Chalk is deeply confined by Palaeogene strata. The Chalk is a maximum of 560 m in thickness.
Lower Greensand Group	The Lower Greensand Group consists of lithologically variable sands and sandstones that are commonly glauconitic or iron-rich, with local clay-rich or sandy limestone beds. Yields of up to about 50 l/sec have been obtained and the water is typically soft and iron rich. It can be up to 250 m in thickness and is utilised at depths of up to 400 m.
Spilsby Sandstone Formation	The Spilsby Sandstone Formation consists of a variably, often poorly cemented quartz sandstone about 24 m thick with alternating thin clays and marls. Yields are typically 25 to 40 l/sec.
Corallian Group	The Corallian Group is present at outcrop in Yorkshire and in the Cotswolds. In Yorkshire it consists of limestones and grits up to about 100 m thick, thinning to about 20 m towards the south of the region, where the limestones are progressively replaced by clay. Here it yields up to 15 l/sec. In the Cotswolds the aquifer is up to 40 m thick and comprises limestones, marls and sandstones. It provides yields of 5 to 10 l/sec, with water quality becoming increasingly saline down dip as the aquifer becomes confined in the Wessex Basin.
Great and Inferior Oolite groups	The Great and Inferior Oolite groups consist of limestones, sands and clays up to 200 m in Somerset, Dorset and the Cotswolds. Yields are typically in the range 5 to 15 l/sec. Limestones in the aquifer become increasingly compact within the sequence down dip and the water rapidly becomes brackish as the aquifers become confined.
Triassic sandstone	Sandstone of Triassic age forms England's second most important aquifer (after the Chalk) and consists of sandstones, conglomerates and marls up to 600 m thick. It can yield up to 125 l/sec of good quality hard to moderately hard water from the upper parts of the aquifer. It is utilised at depths of up to 400 m, but elsewhere groundwater becomes highly saline with depth.
Zechstein Group	The Zechstein Group consists of massive dolomitic and reef limestones with evaporites, mudstones and siltstones and is up to 300 m thick near Durham. Yields range up to 50 l/sec from the upper parts of the aquifer but the water is typically very hard.
Permian sandstone	Sandstone of Permian age consists of sandstones, marls and breccias. In southwest England it reaches 700 m in thickness with yields up to about 15 l/sec. In northwest England it may reach up to 900 m in thickness and yields are up to about 25 l/sec from the upper parts of the aquifer. The water in the Permian sandstone becomes highly saline at depth.
Carboniferous limestone	The Carboniferous limestone is a massive, well-fissured karstic limestone that gives large water supplies of up to 175 l/sec from resurgences in the Mendips and South Wales, and borehole yields of up to 40 l/sec from the upper parts of the aquifer. It can be up to about 800 m in thickness.
Border Group	The Fell Sandstone Formation is part of the Border Group. It consists of friable, quartz-rich sandstones with minor pebbly and silty bands. The Fell Sandstone Formation is up to about 300 m in thickness. Yields are quite variable due to variations in the degree of jointing, but typically less than 10 l/sec from the upper parts of the aquifer.

Table S2. List and description of the major shale units in England and Wales and analysed in this work.

**Major shale**	**Description of major shale unit**
Kimmeridge Clay Formation	The modelled unit consists of the Kimmeridge Clay and Ampthill Clay formations. The Kimmeridge Clay Formation comprises calcareous or kerogen rich or silty and sandy mudstones. It is thought to be a potential prospect for oil and probably biogenic gas (DECC, 2012) because it contains ubiquitous oil-shale beds. Its background total organic carbon (TOC) is up to about 10%, its bituminous beds have TOC values up to 30% and its oil-shales have TOCs reaching 70%. It thickens southwards up to 500 m.
Oxford Clay Formation	The modelled unit consists of the Kellaways, Oxford Clay and Osgodby formations. These are lithologically diverse. They include mudstones, bituminous clays, calcareous siltstones, beds with limestone nodules and local sandstones. DECC (2012) notes that the Oxford Clay Formation is immature for hydrocarbon generation in the English Midlands, but that in the Weald Basin its TOC reaches 7.83% and it lies within the oil window at the basin's depocentre. TOCs average below 2% in the non-bituminous sections but are over 4% in most of the bituminous sections of central England, where they are immature for oil. The Oxford Clay Formation can be up to 185 m in thickness in south Dorset, but is typically 60 m thick.
Lias Group	The Lias Group consists of thick mudstones and clays with alternating bands of thin limestones in the lower part and becoming sandier in the upper part. DECC (2012) states that the Lias Group, though probably lying in the oil window, is immature for shale gas. The Lias Group is the source rock for the Weald Basin petroleum system and for the Wessex Basin, with migration into three different reservoirs at Wytch Farm oil field. The Lias Group can be up to about 700 m in thickness in southern England.
Marros Group	The Marros Group consists of siliceous mudstones and locally quartz rich sandstones and conglomerates. The Marros Group has high gamma-ray responses on geophysical logs, indicating a high organic content (DECC 2012). The Marros Group is up to 750 m in thickness.
Bowland Shale Formation	The modelled unit comprises the Bowland Shale Formation and underlying Hodder Formation. These are fissile organic-rich mudstones, with thin limestones and local sandstone, siltstones, cherts and reef limestones. Andrews (2013) states that the organic content of the Bowland-Hodder shale is typically in the range 1–3%, but can reach 8%. Where they have been buried to sufficient depth for the organic material to generate gas, they have the potential to form a shale gas resource analogous to the producing shale gas provinces of North America. However, central Britain has experienced a complex tectonic history and the rocks have been uplifted and partially eroded at least once since Carboniferous times. Because of this, the present-day depth to the top of the gas window is dependent on the amount of uplift, and may occur significantly shallower than 2900 m. This unit could be up to 5000 m thick.
Upper Cambrian shales	The Upper Cambrian shales are dark grey and black shales with a high organic content, although they are probably over-mature for conventional hydrocarbons (DECC 2012).

Figure S1. Maps indicating location and terminal depth of water wells in BGS's WellMaster database in: a. Permian and Triassic sandstones of northwest England and b. the Chalk Group of Yorkshire and Lincolnshire. c. Histogram showing the depth distribution of water wells in these two regions.

a.

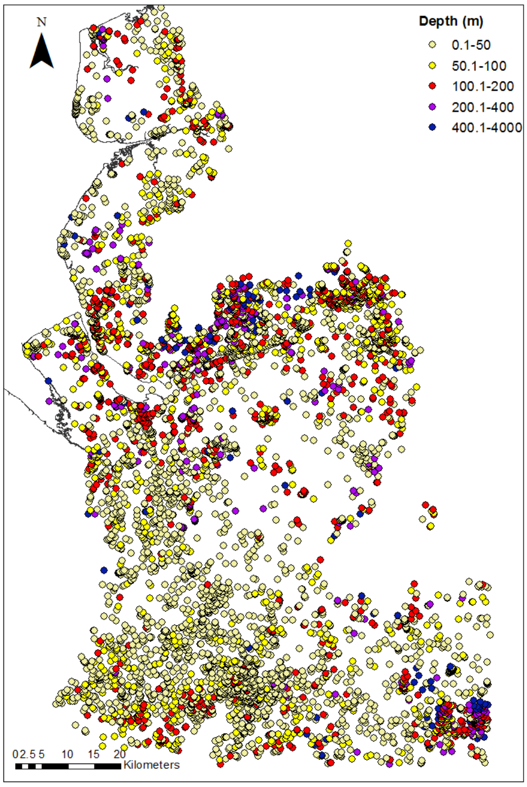

b.

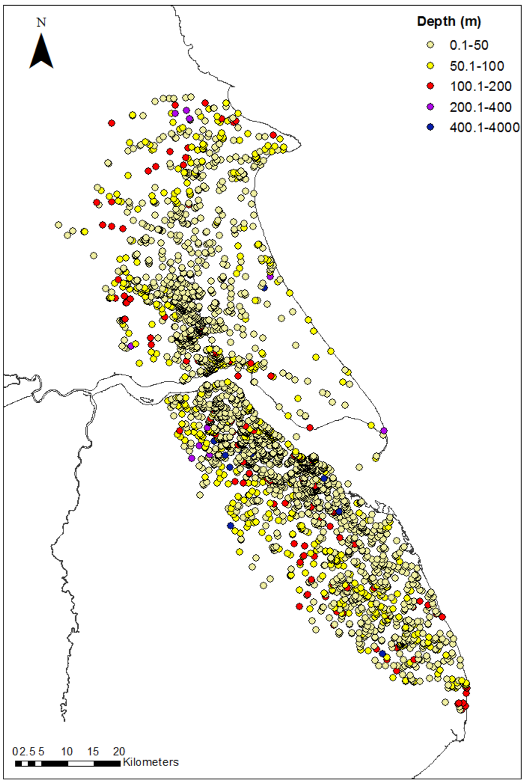

c.

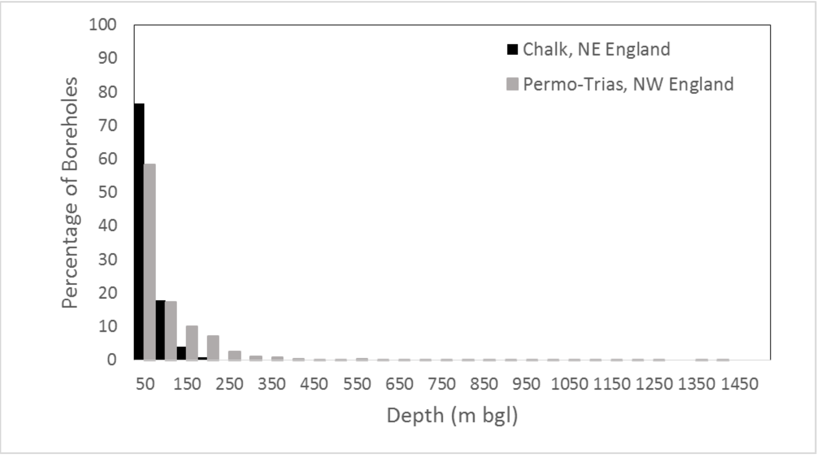

Figure S2a. Maps showing modelled outputs of the depth of the base of the aquifer rock unit. Depth is m above OD.

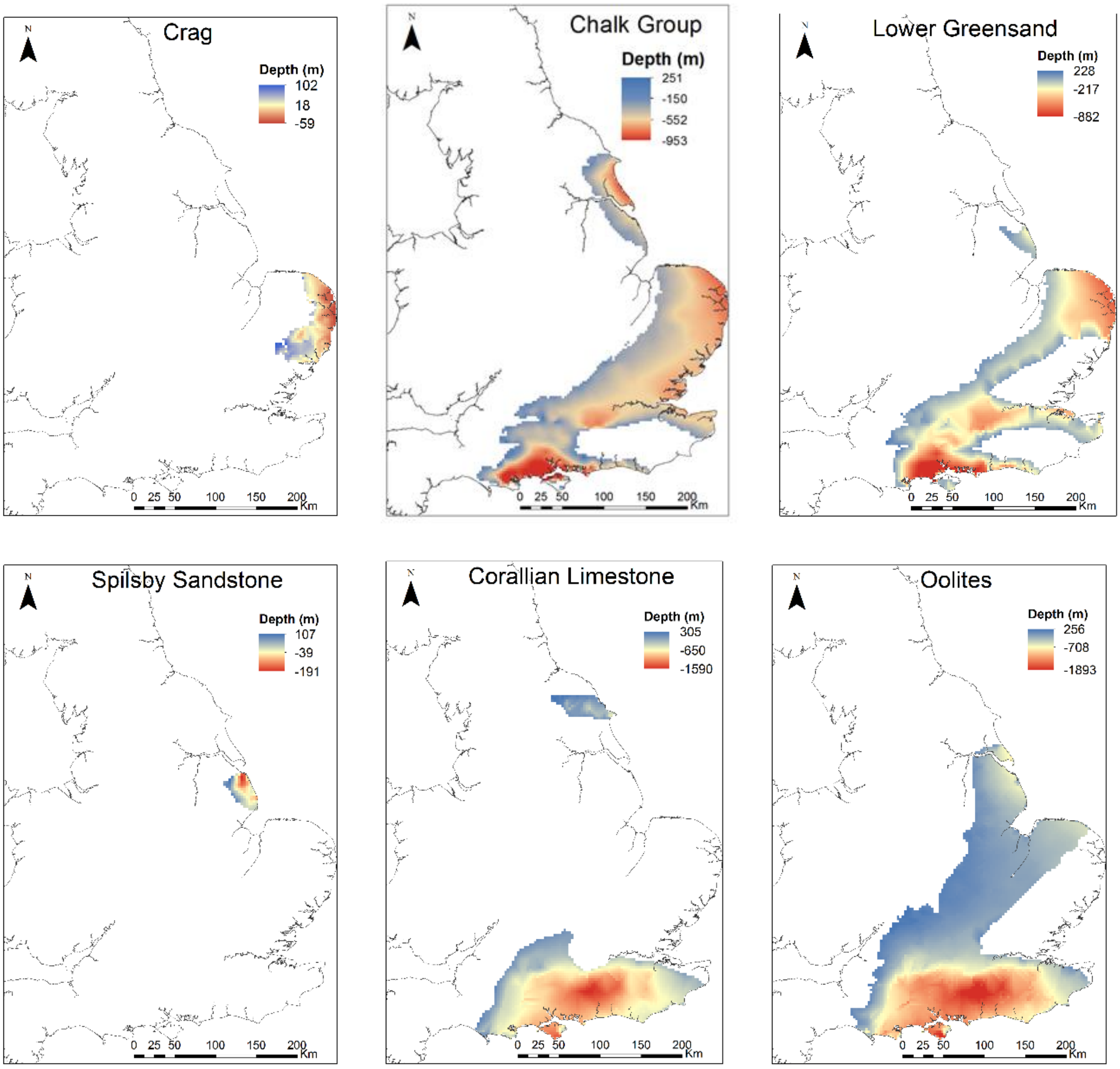

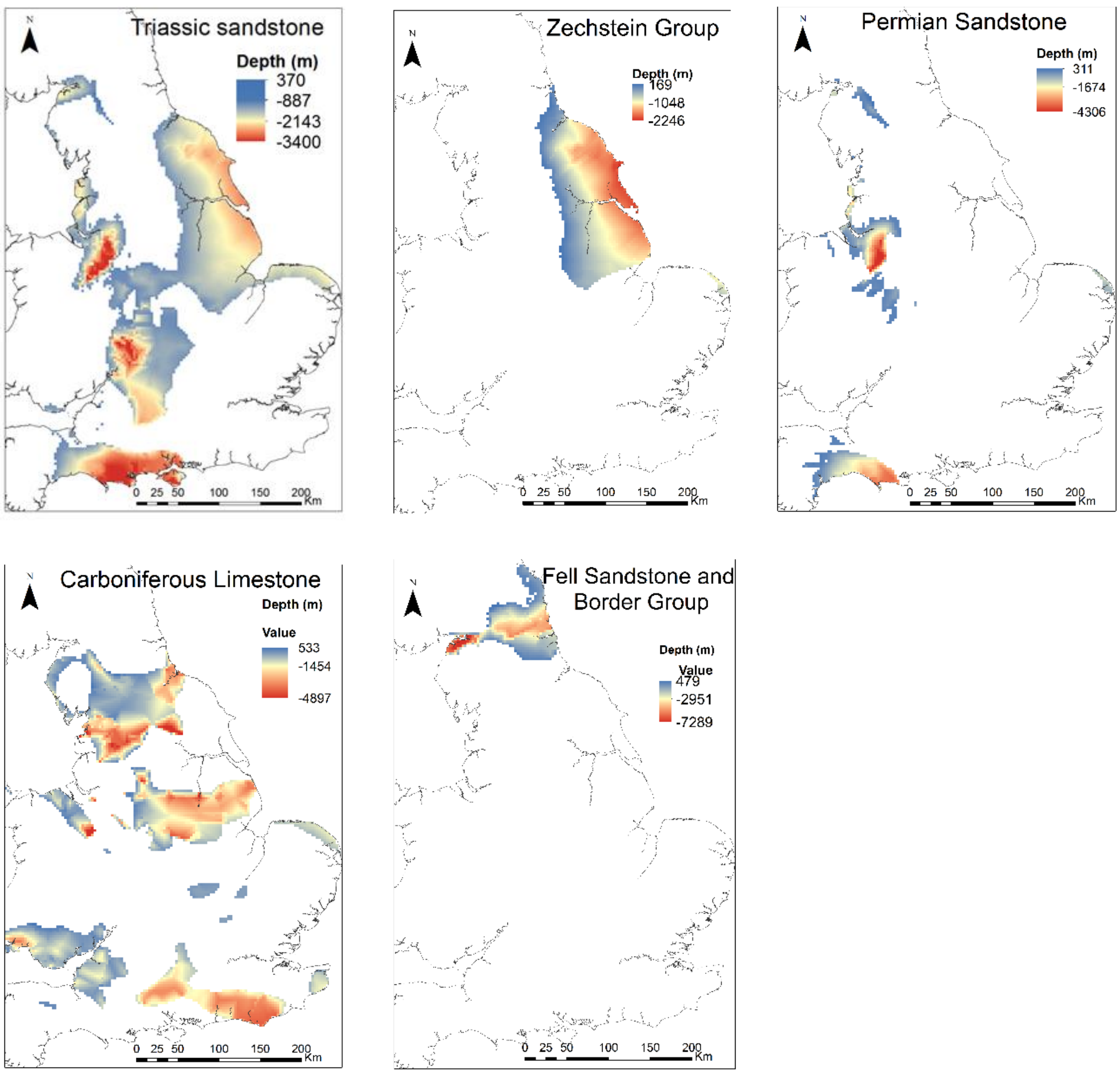

Triassic sandstone
Depth (m)
370
-887
-2143
-3400
0 25 50 100 150 200
Km
Zechstein Group
Depth (m)
169
-1048
-2246
0 25 50 100 150 200
Km
Permian Sandstone
Depth (m)
311
-1674
-4306
0 25 50 100 150 200
Km
Carboniferous Limestone
Depth (m)
Value
533
-1454
-4897
0 25 50 100 150 200
Km
Fell Sandstone and Border Group
Depth (m)
Value
479
-2951
-7289
0 25 50 100 150 200
Km

Figure S2b. Maps showing modelled outputs of the depth of the top of the shale unit. Depth is m above OD.

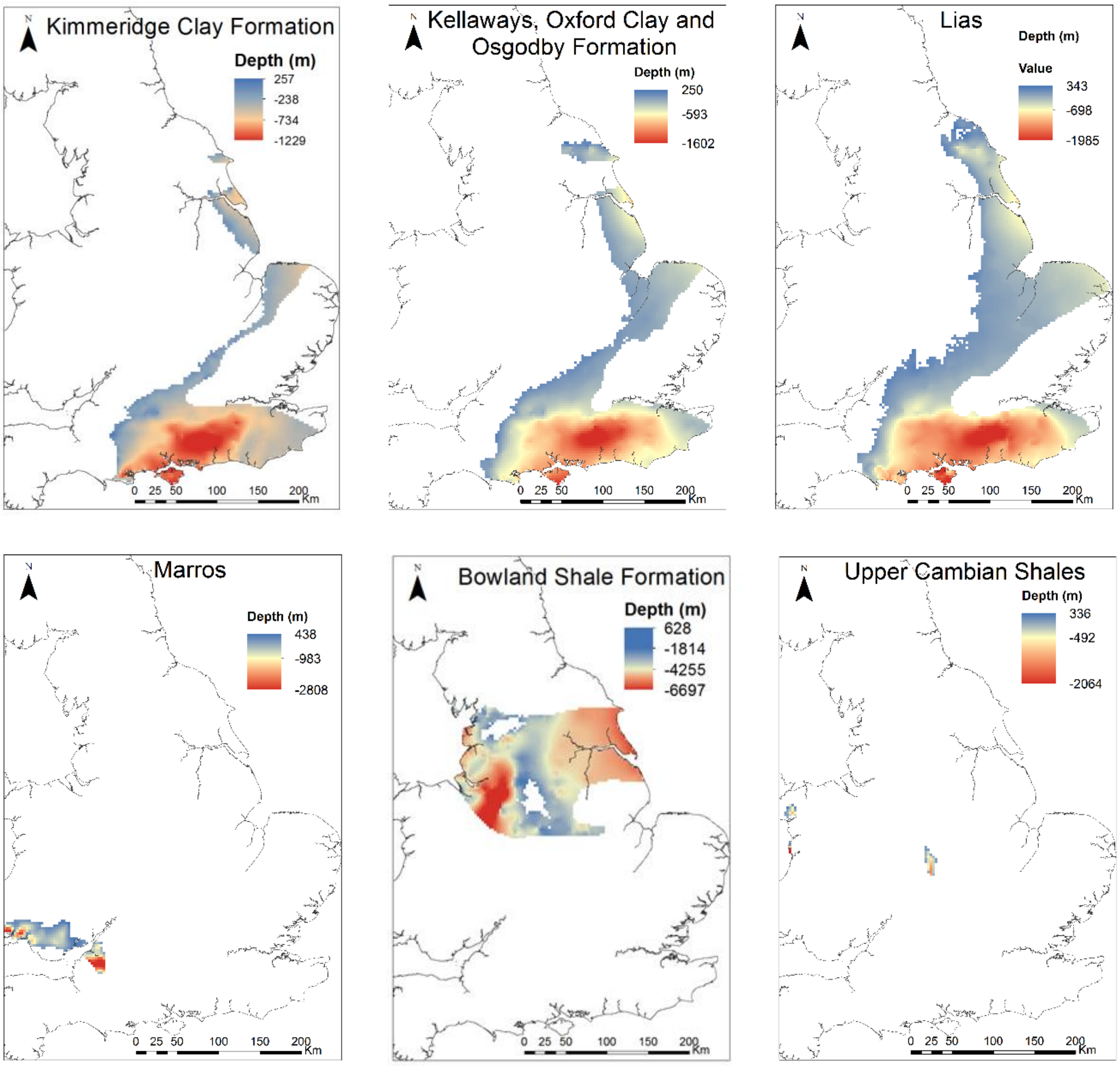

Figure S2c. Maps showing modelled outputs for vertical separation between shales and aquifer (maximum depth 400 m bgl). Contours in vertical separation maps show the areas where the shale formation is > 1000 m bgl.

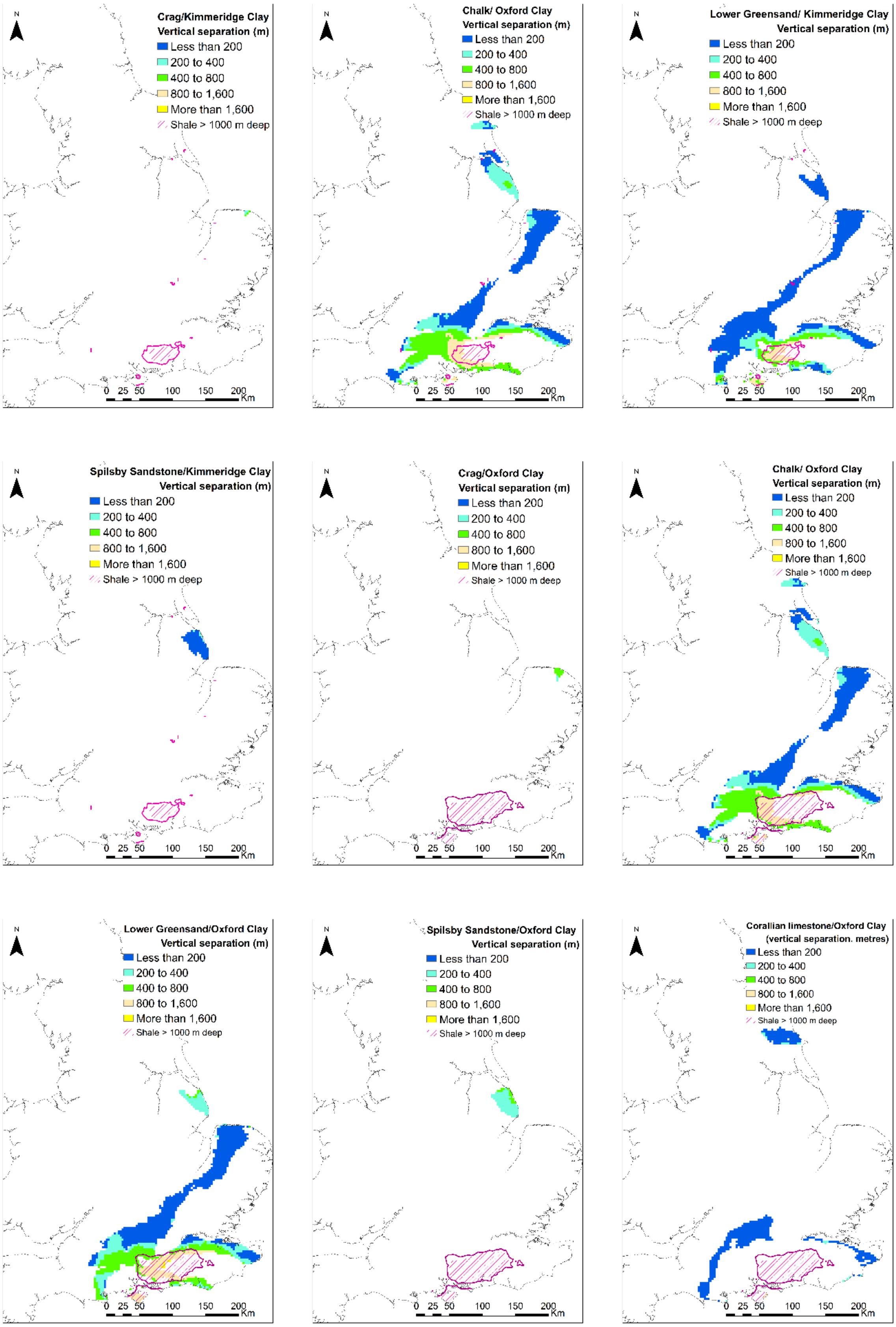

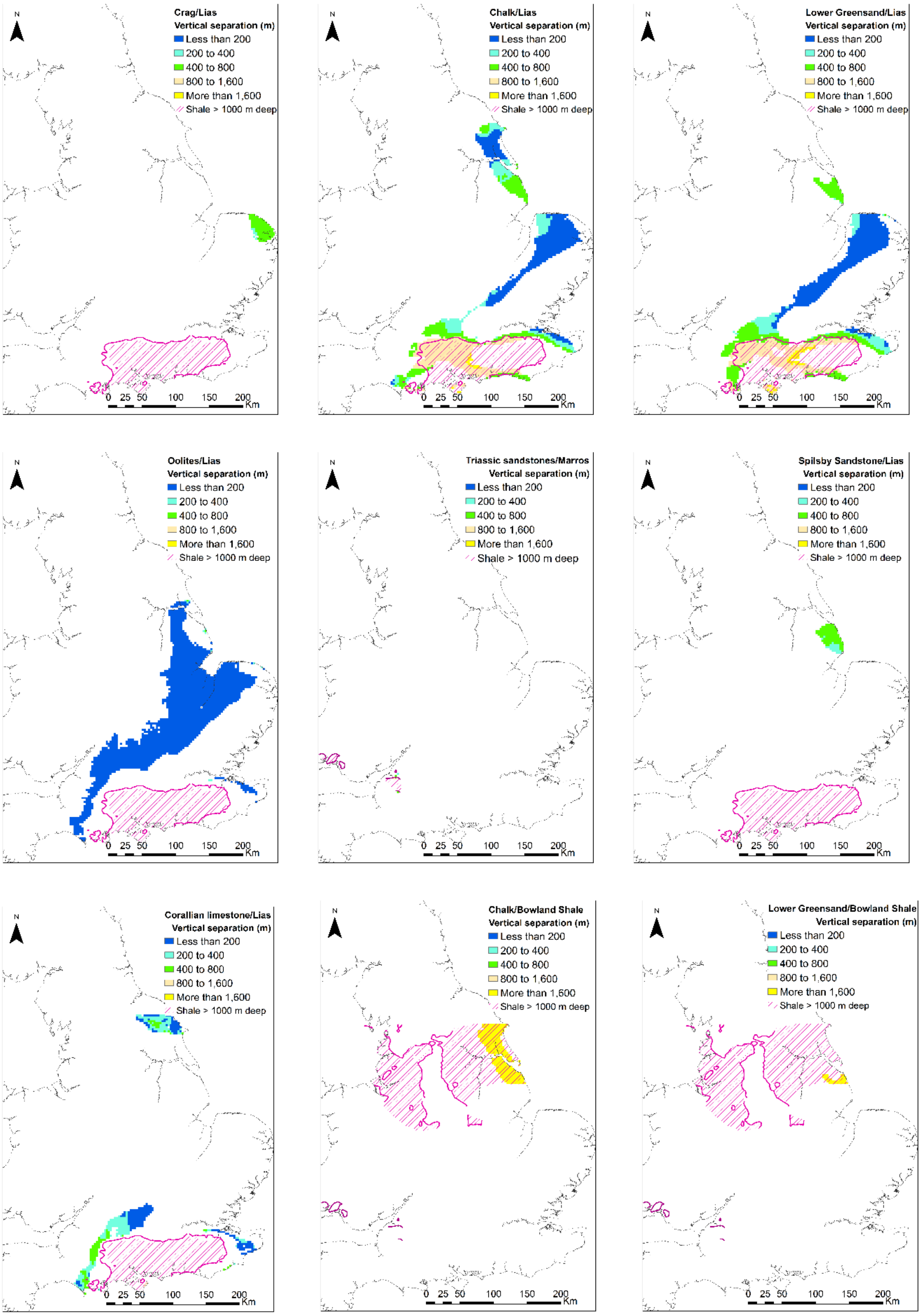

Crag/Lias
Vertical separation (m)
Less than 200
200 to 400
400 to 800
800 to 1,600
More than 1,600
Shale > 1000 m deep
Chalk/Lias
Vertical separation (m)
Less than 200
200 to 400
400 to 800
800 to 1,600
More than 1,600
Shale > 1000 m deep
Lower Greensand/Lias
Vertical separation (m)
Less than 200
200 to 400
400 to 800
800 to 1,600
More than 1,600
Shale > 1000 m deep
Oolites/Lias
Vertical separation (m)
Less than 200
200 to 400
400 to 800
800 to 1,600
More than 1,600
Shale > 1000 m deep
Triassic sandstones/Marros
Vertical separation (m)
Less than 200
200 to 400
400 to 800
800 to 1,600
More than 1,600
Shale > 1000 m deep
Spilsby Sandstone/Lias
Vertical separation (m)
Less than 200
200 to 400
400 to 800
800 to 1,600
More than 1,600
Shale > 1000 m deep
Corallian limestone/Lias
Vertical separation (m)
Less than 200
200 to 400
400 to 800
800 to 1,600
More than 1,600
Shale > 1000 m deep
Chalk/Bowland Shale
Vertical separation (m)
Less than 200
200 to 400
400 to 800
800 to 1,600
More than 1,600
Shale > 1000 m deep
Lower Greensand/Bowland Shale
Vertical separation (m)
Less than 200
200 to 400
400 to 800
800 to 1,600
More than 1,600
Shale > 1000 m deep
N
0 25 50 100 150 200 Km

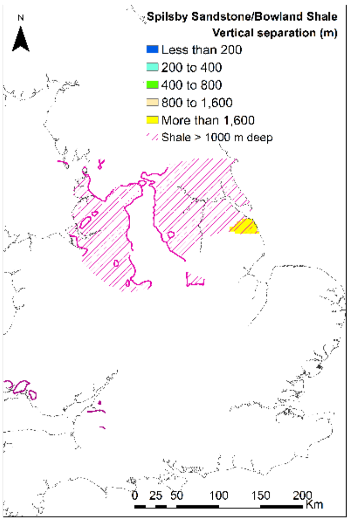
N
Spilsby Sandstone/Bowland Shale
Vertical separation (m)
Less than 200
200 to 400
400 to 800
800 to 1,600
More than 1,600
Shale > 1000 m deep
0 25 50 100 150 200
Km

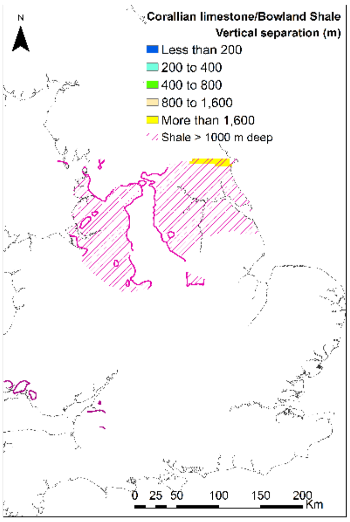
N
Corallian limestone/Bowland Shale
Vertical separation (m)
Less than 200
200 to 400
400 to 800
800 to 1,600
More than 1,600
Shale > 1000 m deep
0 25 50 100 150 200
Km

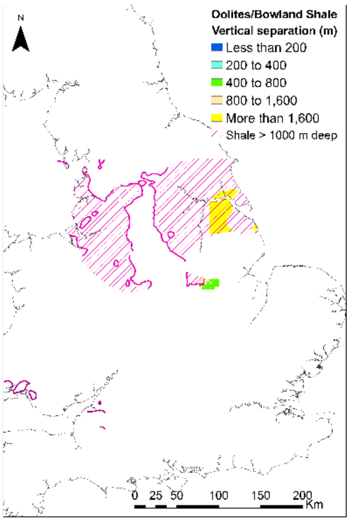
N
Oolites/Bowland Shale
Vertical separation (m)
Less than 200
200 to 400
400 to 800
800 to 1,600
More than 1,600
Shale > 1000 m deep
0 25 50 100 150 200
Km

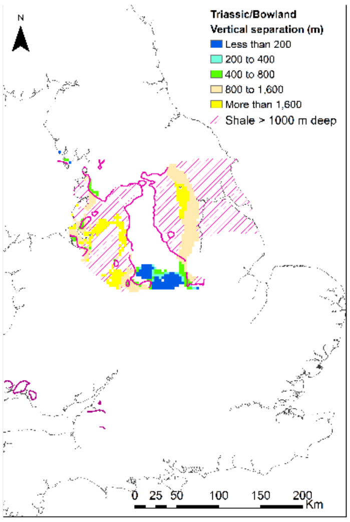
N
Triassic/Bowland
Vertical separation (m)
Less than 200
200 to 400
400 to 800
800 to 1,600
More than 1,600
Shale > 1000 m deep
0 25 50 100 150 200
Km

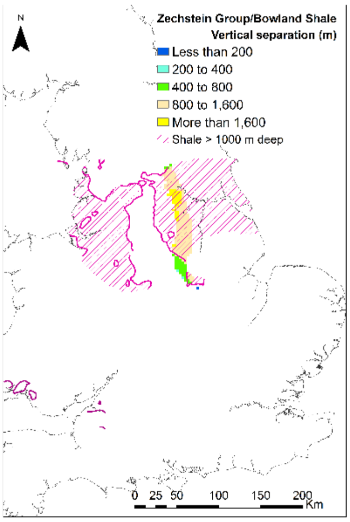
N
Zechstein Group/Bowland Shale
Vertical separation (m)
Less than 200
200 to 400
400 to 800
800 to 1,600
More than 1,600
Shale > 1000 m deep
0 25 50 100 150 200
Km

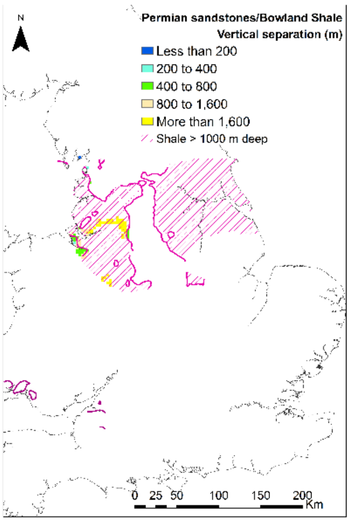
N
Permian sandstones/Bowland Shale
Vertical separation (m)
Less than 200
200 to 400
400 to 800
800 to 1,600
More than 1,600
Shale > 1000 m deep
0 25 50 100 150 200
Km

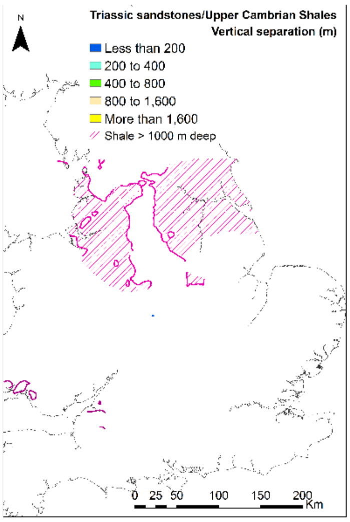
N
Triassic sandstones/Upper Cambrian Shales
Vertical separation (m)
Less than 200
200 to 400
400 to 800
800 to 1,600
More than 1,600
Shale > 1000 m deep
0 25 50 100 150 200
Km

Figure S3.Comparison between a. map of the top of the Bowland Shale Formation (Craven Group) (present study) with b. detailed map of the Bowland-Hodder Shale formations (after Andrews, 2013). A common colour ramp was applied to indicate spatial variations in depth. The Bowland, Cheshire and Cleveland basins are all well-resolved in the mapping produced by the present study as are the Widmerpool Trough, the East Midlands Shelf and the area of eroded Bowland Shale Formation to the east of the Cheshire Basin.

a

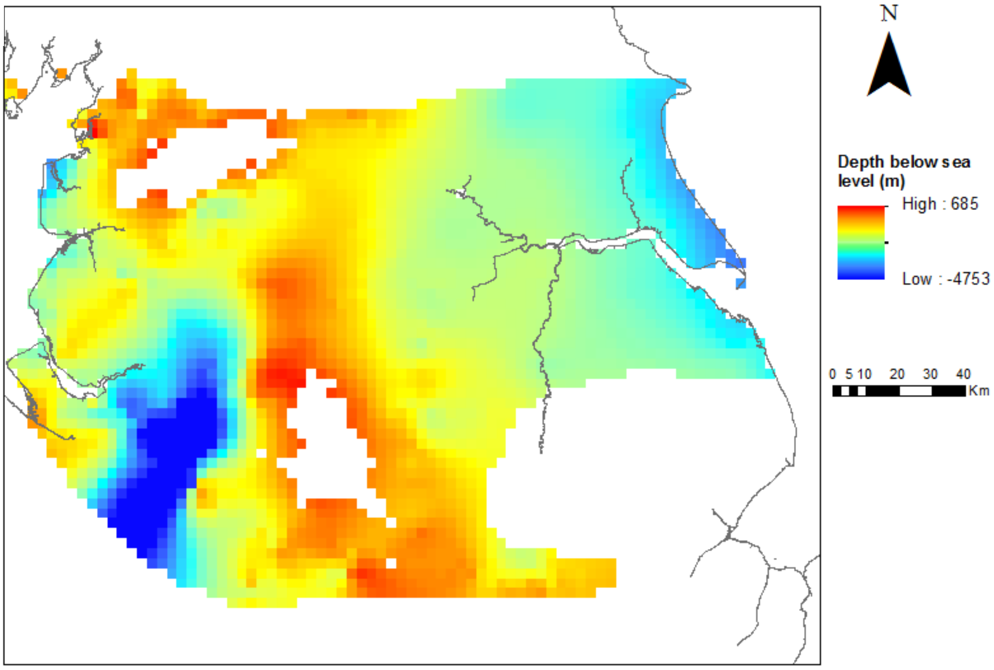

b.

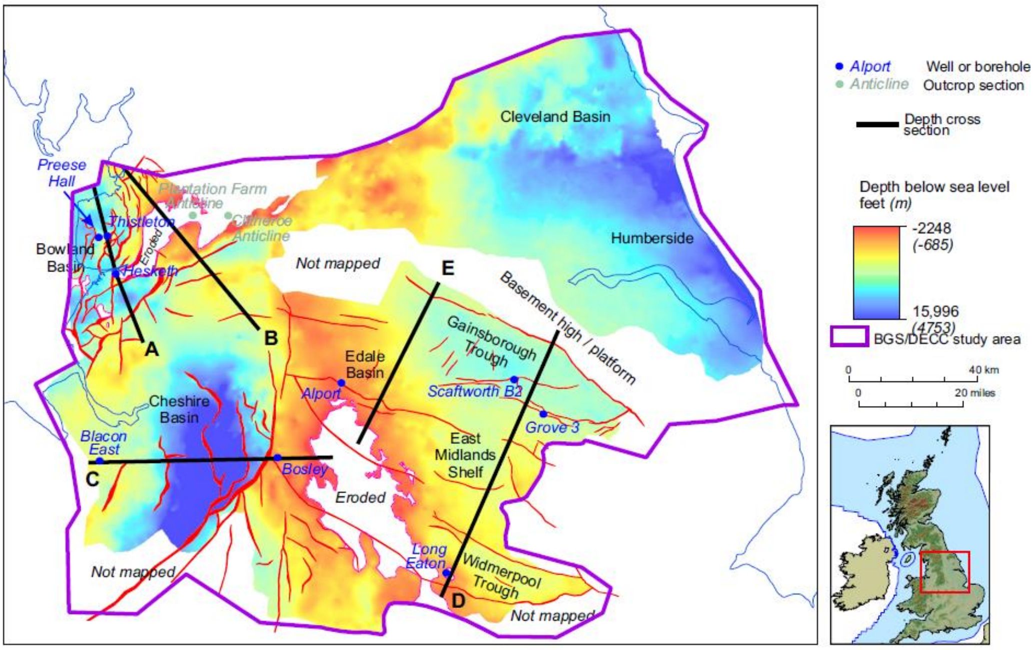

ESM References

Andrews IJ (2013) The Carboniferous Bowland Shale-gas study: geology and resource estimation, British Geological Survey for the Department of Energy and Climate Change, London, UK.

DECC (2012) The unconventional hydrocarbon resources of Britain's onshore basins - shale-gas, Department of Energy and Climate Change, London, UK.
